# Recent Advances Towards Selenium Nanoparticles: Synthetic Methods, Functional Mechanisms, and Biological Applications

**DOI:** 10.3390/foods14213640

**Published:** 2025-10-24

**Authors:** Lulu Geng, Linling Li, Xuening Sun, Shuiyuan Cheng, Jiangling He

**Affiliations:** 1 National R&D Center for Se-rich Agricultural Products Processing, Wuhan Polytechnic University, Wuhan 430023, China; genglulu0905@163.com (L.G.); 15188571791@163.com (X.S.); s-y-cheng@sina.com (S.C.); 2 School of Modern Industry for Selenium Science and Engineering, Wuhan Polytechnic University, Wuhan 430023, China

**Keywords:** selenium nanoparticle, synthetic method, functional mechanism, biological application

## Abstract

The exceptional physicochemical properties of selenium nanoparticles (SeNPs) have led to their widespread development. The function of SeNPs is significantly influenced by their shape and particle size, which are in turn determined by the applied synthesis methods. This work presents a critical and comparative analysis of physical, chemical, and biosynthetic methods. The key point is to elaborate on how different methods precisely regulate the particle size, morphology, and stability that are crucial to their functional efficacy. This work emphasizes the importance of creating standardized protocols for characterizing SeNPs in order to make meaningful comparisons between the effectiveness of various studies. We further elucidate the underlying mechanisms of SeNPs’ anti-tumor, antioxidant, and antibacterial activities. A key novelty of this work lies in its systematic construction of a bridge between the synthesis, properties, functions, applications, and translational potential and its provision of a critical assessment. Finally, the review identifies and summarizes the principal challenges hindering clinical and commercial translation, including the imperative for standardized toxicological evaluation, scalable synthesis, and regulatory alignment.

## 1. Introduction

Selenium (Se) is an essential micronutrient for humans and animals [1], and it is involved in many important life processes, such as anti-cancer activity, anti-oxidation property, enhancing human immunity, antagonizing harmful heavy metals, and regulating protein synthesis [2]. The bioavailability and toxicity of selenium are highly dose-dependent, with a narrow window between necessity and excess, which has spurred interest in developing safer and more efficacious selenium forms [3,4]. The advent of nanotechnology has opened new avenues, with selenium nanoparticles (SeNPs) emerging as a promising functional material due to its superior biocompatibility and lower toxicity compared to its organic and inorganic counterparts [5]. SeNPs have shown brilliant prospects in various research fields [2,6,7]. While various physical, chemical, and biosynthetic approaches have been developed to fabricate SeNPs, a systematic understanding of how these methods precisely control nanoparticle properties and, consequently, their functional performance, remains fragmented [8,9]. Smaller SeNPs may have greater biological activity, with the biological activity decreasing with increasing nanoparticle size [1]. Nanoparticles are unstable, so SeNPs are usually modified to give them stable physicochemical properties. Zhang et al. modified SeNPs using a coupling of *Grifola frondosa* polysaccharides and gallic acid, and prepared SeNPs that could be stored in a dark storage environment at 4 °C for 70 days while still maintaining stability [10]. The synthesis method has a significant impact on the properties of SeNPs. Takahashi et al. found that SeNPs biosynthesized using *Escherichia coli* K-12 have a lower absorption capacity and lower nutrient utilization than chemically synthesized SeNPs in the intestines [11].

**Figure 1:**
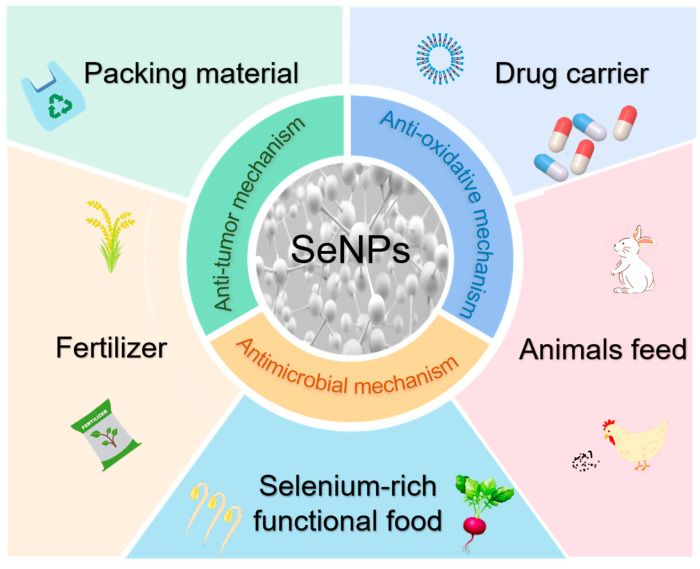
The typical functional effects and biological applications of SeNPs (Some schematic elements were downloaded from “Vecteezy” website; the elements downloaded from this website are free).

Owing to their unique size and surface chemistry, SeNPs exhibit multiple biological activities, including anti-tumor, antibacterial, and antioxidant properties [12,13,14,15,16]. These characteristics also determine the wide application of SeNPs. SeNPs have occupied pivotal positions in packaging materials [17], functional foods [18], fertilizers [19], animal feed [20], drug carriers [21], and other aspects [22,23]. In the fields of agriculture and food, SeNPs can not only be added as functional components to staple foods, such as rice, providing selenium supplementation for the human body while improving crop yield and quality [24], but also can be used to inhibit rice diseases [25] and enhance the tolerance of crops to heavy metal stress [26]. In the field of medicine and health, SeNPs can be applied in oxidative stress treatment, and to accelerate cell metabolism and to inhibit osteoclastogenesis, and the effective treatment of bone diseases [27] is also noted. SeNPs also play a pivotal role in poultry nutrition [28,29]. Mohammadi et al. explored the daily weight gain of broiler chickens over a period of 42 days by adding different sources of selenium, such as sodium selenite (Na_2_SeO_3_), selenium-enriched yeast, and SeNPs, to the feeds of poultry. Compared to the other forms of selenium, SeNPs improved the selenium content of the chicken breast meat and the humoral immune properties of broilers, based on the significant weight gain in the broilers [30]. Moreover, as biological nanomaterials, SeNPs have important physiological functions and a wide range of pharmacological effects. Therefore, SeNPs have received extensive attention in drug delivery systems due to their excellent biocompatibility and drug delivery capacity [31]. Gong et al. prepared β-cyclodextrin-folate-modified SeNPs and used SeNPs as drug carriers for paclitaxel, and found that the nanosystems increased the selectivity for normal and cancer cells, thus achieving the target-specific recognition of MCF-7 in cancer cells and enhancing the toxicity of cancer cells [32]. The typical functional effects and biological applications of SeNPs are presented in Figure 1.

As a new type of nanomaterial, SeNPs show great potential and practical significance in many fields owing to their distinctive physicochemical characteristics and biological activities. This work summarizes and reviews the synthesis methods of SeNPs, their functional activity mechanisms, and their applications in packaging materials, functional foods, fertilizers, animal feed, and as drug carriers. In addition to integrating into the existing literature, this review also provides forward-looking perspectives on the current challenges related to toxicity, long-term stability, scalable production, and regulatory barriers. We aim to pave the way for the rational design and further utilization of SeNPs by highlighting these underexplored yet critical aspects.

## 2. Preparation Methods of SeNPs

### 2.1. Physical Methods for the Synthesis of SeNPs

**Figure 2:**
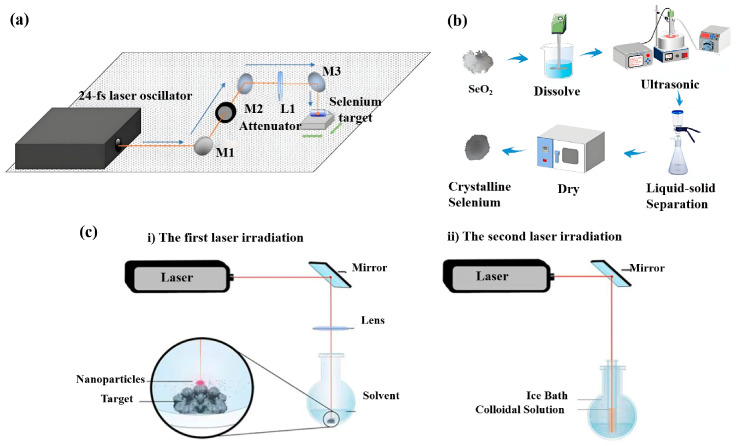
Typical physical methods for the preparation of SeNPs. (**a**) Liquid pulsed laser ablation technique employed to prepare SeNPs, adapted with permission from the copyright 2022, Springer Nature [33]. (**b**) Reduction of selenium dioxide using ultrasound induced selenium dioxide to obtain SeNPs with different morphologies, adapted with permission from the copyright 2023, Elsevier [35]. (**c**) SeNPs irradiated twice using a laser to obtain spherical selenium nanocolloidal solutions, adapted with permission from the copyright 2020, Elsevier [34].

Haro-Poniatowski et al. synthesized nanoparticles via pulsed laser ablation in liquids, utilizing high-purity selenium targets immersed in diverse solvents. The red nanoparticles were evenly distributed in the solution with a particle size between 5 and 200 nm [33] (as shown in Figure 2a). In order to prepare SeNPs, Geoffrion et al. also used the pulsed laser ablation technique in liquids (as shown in Figure 2c). The technique promotes electrostatic repulsion through the charge on the surface of the nanoparticles to prevent the SeNPs from aggregating, using a laser to irradiate the selenium particles twice. Then, the spheroidal SeNPs colloidal solution can be obtained [34]. In addition, ultrasonic processing is often used to prepare SeNPs. Ultrasonic waves produce the cavitation effect. When the cavitation bubble bursts, it produces high-speed micro-jets and shocks in the local area, causing a large impact on the surface of SeNPs, and dispersing the particles into more crystal cores. Yang et al. used ultrasound to induce selenium dioxide reduction, which affects the crystallization process of selenium [35] (as shown in Figure 2b). By adjusting the ultrasonic parameters and the reduction temperature, SeNPs with various shapes (selenium nanorods, selenium nanotubes, and selenium nanospheres) can be obtained with different shapes. The microwave-assisted method can also be used for the synthesis of SeNPs. Mellinas et al. used *Theobroma cacao* L. bean shell extract as a reducing agent and stabilizer to add Na_2_SeO_3_ for the synthesis of SeNPs, using the action of the microwave. The SeNPs obtained can be stable for 55 days at 4 °C and have excellent antioxidant properties [36]. Yu et al. used L-asparagine to reduce H_2_SeO_3_ using a microwave and found that a longer radiation time during synthesis can increase the concentration of SeNPs and can have a significant impact on their diameter and morphology. After 15 min of microwave treatment, the morphology of SeNPs changes from amorphous spherical nanospheres to triangular nanotubes, so a simple and rapid synthesis method was developed to prepare SeNPs with different shapes and antioxidant capacities [37]. Therefore, physical methods can well control the size and form of SeNPs, by controlling radiation time, adjusting power and temperature, etc., but their conversion potential is often limited by economic factors and scalability (The synthesis method is detailed in Table S1).

### 2.2. Chemical Methods for the Synthesis of SeNPs

**Figure 3:**
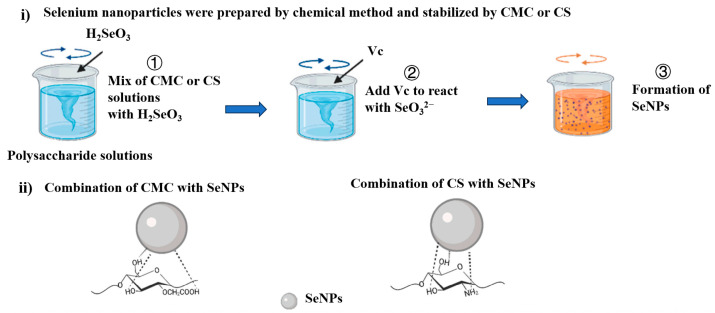
Typical chemical method for the preparation of SeNPs. Schematic representation of SeNPs binding to polysaccharides, adapted with permission from the copyright 2022, Elsevier [40].

Chemical reduction represents the predominant route taken when synthesizing SeNPs, wherein inorganic selenium sources like Na_2_SeO_3_ and selenium dioxide are reduced in the presence of stabilizing agents. Boroumand et al. reduced Na_2_SeO_3_ with ascorbic acid to prepare SeNPs and characterize them by various methods. The formed SeNPs were spherical, with an average diameter of 66.55 ± 8.46 nm, and had strong cytotoxicity for fibroblasts [38]. During the formation process of SeNPs, the initial formation often yields small particles with high surface energy, driving them to aggregate to achieve a lower energy state. However, as this aggregation occurs, SeNPs increase in size, surface free energy decreases, and biological activity weakens. The particles are small, but the surface free energy is large. The aggregation of particles can make the energy of the system very low, thus making the stable existence of the SeNPs aggregate. Therefore, a stabilizing agent needs to be added in the process of SeNPs generation, so that the SeNPs can stably exist and to improve their dispersibility. The common stabilizers are sodium chitosan [39], carboxymethyl cellulose [40] (as shown in Figure 3), carrageenan [41], and other saccharides. Glycerol [42], polyethylene glycol [37], etc., can also be used. In order to make the SeNPs’ particle size small enough, Jiang et al. dissolved Na_2_SeO_3_ in glycerol and used glucose as a reducing agent to prepare SeNPs and nanorods, proposing a controllable and rapid method for preparing small SeNPs with a size less than 6 nm. The use of glycerol and glucose is compatible with human cells, more environmentally friendly, and can be widely used [42]. The chemical synthesis of SeNPs is not only costly but also technically challenging. A major drawback is the frequent use of biologically incompatible reagents and the potential for toxic chemical residuals, which can induce cytotoxicity and limit practical implementation. Additionally, the potential environmental hazards associated with chemical precursors and by-products present sustainability issues, while residual contaminants may hinder the subsequent processing and practical implementation of the resulting nanoparticles. To address these issues, there is a growing trend towards employing greener chemical agents, such as natural polymers and sugars, which can act as both reductants and stabilizers (The synthesis method is detailed in Table S2).

### 2.3. Biosynthesis Methods for the Synthesis of SeNPs

#### 2.3.1. Microbial Synthesis Method for the Synthesis of SeNPs

**Figure 4:**
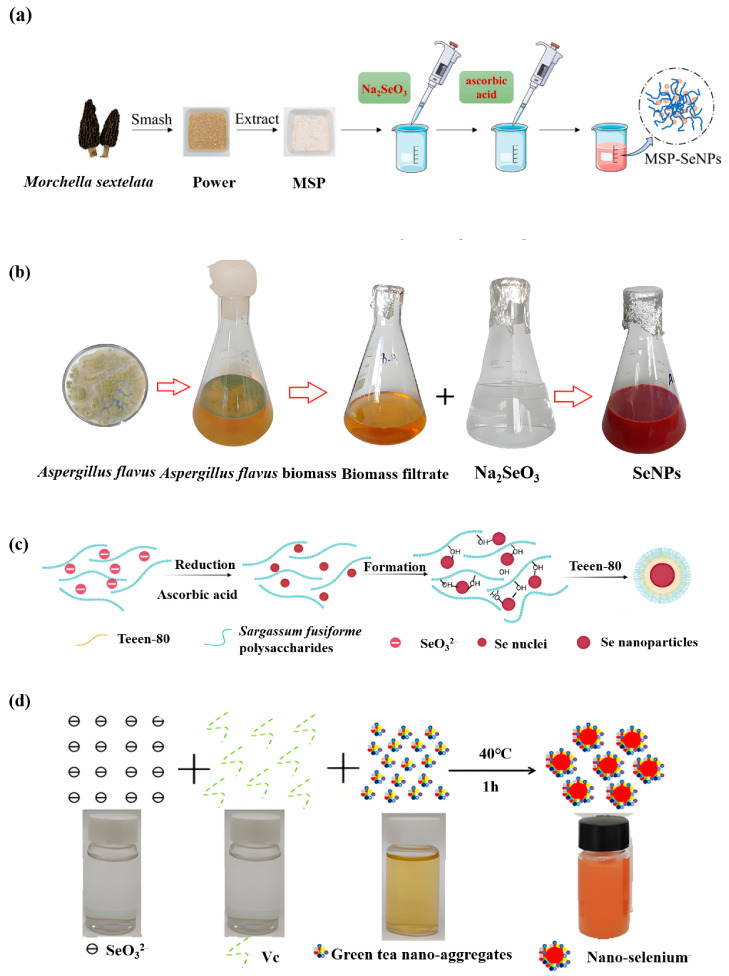
Typical biological method for the preparation of SeNPs. (**a**) Preparation of SeNPs using polysaccharides from *Morchella sextelata* as stabilizers, adapted with permission from the copyright 2022, Elsevier [45]. (**b**) Biosynthesis of SeNPs using *Aspergillus flavus*, adapted with permission from the copyright 2024, Elsevier [48]. (**c**) Preparation of spherical SeNPs using *Sargassum* polysaccharide and tween-80, adapted with permission from the copyright 2024, Elsevier [54]. (**d**) Preparation of amorphous SeNPs using green tea as a template, adapted with permission from the copyright 2020, Elsevier [57].

Metabolites or some enzymes produced by microorganisms during growth and metabolism can reduce inorganic selenium [43]. These natural, bio-active molecules can also play a certain role in modifying selenium, so that SeNPs can exist stably. *Saccharomyces cerevisiae* can effectively reduce selenium ions to some extent. Faramarzi et al. added selenate to the medium of *Saccharomyces cerevisiae*. The color of the sample then changed during the incubation process and became dark red, indicating the formation of SeNPs. A variety of probiotics can tolerate a certain concentration of Na_2_SeO_3_ [44]. *Morchella sextelata* polysaccharide (MSP) can be used to improve the function of SeNPs. Shi et al. used MSP as a stabilizer to reduce Na_2_SeO_3_ with ascorbic acid to prepare SeNPs. MSP-SeNPs are spherical and amorphous particles with a size of 72.07 ± 0.53 nm [45] (as shown in Figure 4a). In addition, *Bacillus subtilis* T5 can produce selenium polysaccharides and SeNPs, and the biosynthesized SeNPs have high stability and a small particle size, showing good probiotic potential [46]. A variety of probiotics can generally tolerate a certain concentration of selenite. Li et al. screened a Se (IV)-resistant *Lactobacillus paralimentarius* strain JZ07 with strong selenium conversion ability. These have the latent capacity to be used as selenium supplements [47]. Mohammed et al. used Aspergillus flavus to biosynthesize SeNPs with sizes ranging from 28 nm to 78 nm, and explored their antibacterial, antiviral, antioxidant and anti-tumor activities, which have no hemolytic activity on human red blood cells, and show great safety in application [48] (as shown in Figure 4b). Therefore, compared with other methods, the microbial synthesis of SeNPs has many advantages, such as mild conversion conditions, no specific equipment, good safety levels, and environmental protection. The research on the microbial synthesis of SeNPs is not limited to bacteria and fungi; it also includes protozoa. A simple, economical, and environmentally friendly method for SeNPs synthesis using *Tetrahymena thermophilus* SB 210 was studied by Cui et al.; electron microscopy, energy dispersion X-ray spectrum, and Raman spectroscopy show that red spherical SeNPs, of 50 to 500 nm in diameter, are synthesized in vivo [49].

#### 2.3.2. Plant Synthesis Method for the Synthesis of SeNPs

Plants absorb environmental inorganic selenium during their growth and convert it into organic selenium. Alvi et al. synthesized SeNPs using a simple green method to analyze the antibacterial activity of citrus fruit extracts based on SeNPs. *Citrus limon* and *Citrus paradisi* were used as green sources [50]. Green-synthesized SeNPs are more widely used. Salem et al. synthesized SeNPs using pomegranate peel extract; the synthesized spherical nanoparticles had an average diameter of 9.41 nm and had an effective fungicidal potential against *Penicillium digitatum*, and the coating solution based on the nanoparticles reduced the signs of infection in oranges and prevented fungal invasion [51]. Green synthesis of SeNPs have the diversity of biologically active molecules, and Jeevanantham et al. used a sustainable and environmentally friendly method of selenium nano crystals, with *Coccinia grandis* fruit extract as capping agent and stabilizer added to Na_2_SeO_3_ solution, with the solution then placed into a microwave reactor for reaction. SeNPs exhibited both antioxidant and antibacterial properties, demonstrating potent bactericidal efficacy against common infectious skin disease pathogens such as *Escherichia coli*, *Bacillus cereus*, and *Staphylococcus aureus* [52]. The polysaccharides in plants have good biocompatibility and are considered a suitable choice for the green synthesis of SeNPs [53]. Chen et al. prepared spherical SeNPs with a particle size of about 50 nm by using polysaccharides of *Sargassum* as a modifier and tween-80 as a stabilizer. The particle size and PDI of the nanoparticles also increased with the increase in temperature. The SeNPs prepared under optimal conditions had good stability and could inhibit tumor proliferation [54] (as shown in Figure 4c). Zhang et al. obtained an inulin fructan with anti-tumor activity from dandelion and mixed it with Na_2_SeO_3_ to prepare the dispersed and stable spherical SeNPs. In vivo anti-tumor experiments have been carried out on zebrafish, and their anti-tumor activity has been confirmed [55]. Sun et al. prepared hawthorn polysaccharide SeNPs by the HNO_3_-Na_2_SeO_4_ method. The average particle size of the SeNPs system was 137.85 nm, and the free radical scavenging rates of DPPH and ABTS were concentration-dependent. Compared with hawthorn polysaccharide, SeNPs could significantly improve the antioxidant activity of hawthorn polysaccharide [56]. Ye et al. used green tea as a template to prepare amorphous SeNPs with a diameter of 50 nm and high electrostatic stability (−41.25 mV), which were characterized from various aspects and showed strong inhibition on liver cancer cells [57] (as shown in Figure 4d). Despite its eco-friendliness and efficacy, the plant-mediated synthesis method faces challenges in scalability and reproducibility, and the specific functions of key phytochemicals should be deeply explored in the future (The synthesis method is detailed in Table S3).

### 2.4. Other Methods for Synthesis SeNPs

Some scholars have adopted a combination of two methods to prepare SeNPs, such as the combination of physical and chemical methods, or the combination of biological and chemical methods. In the study by El-Batal et al., the combined chemical method with the physical method, and the combined biological method with physical method are used for synthesizing SeNPs. They use a mixture of various natural polysaccharides and a solution of selenium dioxide, or ferment the water extract of fenugreek with *Pleurotus ostreatus*, and then apply gamma radiation until the red SeNPs are produced. The effects of different radiation sources (gamma radiation, ultraviolet radiation, and visible light) on the generation of SeNPs are also studied, and the result is that γ-ray radiation has a better effect on the synthesis of SeNPs [58]. El-Sayed et al. successfully synthesize SeNPs through solid state fermentation rapidly and economically. Furthermore, they also study the promoting effect of γ irradiation on SeNPs. Exposing fungal spores to 1000 Gy gamma rays increases the production of SeNPs about 5-fold. According to the MTT assay, SeNPs inhibit cell proliferation with 50% inhibitory concentrations for normal human melanocytes, with human breast cancer and liver cancer cell lines being 45.21, 61.86, and 200.15 µg/mL, respectively [59]. The combination of two methods when preparing SeNPs has the advantages of being fast, simple, green, and high yield. While hybrid methods hold great promise for SeNPs production, they also add significant complexity to process optimization and scaling. Parameters such as radiation dose, biological extract concentration, and reaction sequence must be carefully controlled to ensure consistent output. Nevertheless, the integration of diverse synthesis techniques represents a forward-looking direction for producing high-quality SeNPs, effectively bridging the gaps between scalability, environmental friendliness, and functional excellence (The synthesis method is detailed in Table S4).

### 2.5. Standardization Challenges in SeNPs Synthesis

The preparation of SeNPs has evolved through various techniques, including physical, chemical, and biological synthesis methods [60,61,62]. However, significant differences in particle size, morphology, surface chemistry, and the capping agents used pose serious challenges for the direct comparison across different studies [63,64]. Therefore, understanding and precisely controlling these key parameters is essential for achieving reproducible synthesis and fair performance evaluation. Typically, the choice of synthesis method directly determines the initial characteristics of SeNPs. Physical methods can control the particle size and morphology of SeNPs by adjusting factors such as radiation time, power, and temperature. Chemical reduction methods can produce well-dispersed, spherical SeNPs by precisely controlling precursor concentration, and reducing agent concentration, feed ratio, reaction temperature, and pH. In contrast, microbial synthesis tends to yield amorphous or quasi-spherical SeNPs with a broader size distribution and more complex regulation. On the other hand, natural capping using biomacromolecules (such as proteins and polysaccharides) imparts excellent colloidal stability and inherent biocompatibility to the particles. In the standardized comparison of SeNPs produced by different methods, it is essential to regulate their size and morphology through controlled synthesis and to perform detailed characterization. This entails determining key properties such as particle size, polydispersity index, and zeta potential, along with analyzing morphology, surface chemistry, and capping agents to establish a complete physicochemical profile. Only on this basis can functional comparisons of SeNPs across different studies be scientifically meaningful, thereby advancing the transition of SeNPs from the laboratory to practical applications.

## 3. The Function Mechanisms of SeNPs Activities (Figure 5)

### 3.1. The Mechanism of Anti-Tumor Function

Tumor initiation and progression are studied by the random emergence and functional selection of somatic DNA mutations. Compared with traditional organic selenium and inorganic selenium, SeNPs have the advantages of high safety, good absorption, diverse biological activities, and drug-carrying capacity. Exploring their mechanisms of function has become a research hotspot in selenium nutrition science and nanodrug carrier studies. Bao et al. applied selenite treatment to cancer cell H157, after TEM identification, and found that it forms endogenous SeNPs. By conducting the genome expression analysis of H157 cells formed by endogenous SeNPs, they found that mitochondrial RNA is significantly downregulated, proving the existence of the mitochondrial function inhibition pathway [65]. Liu et al. prepared lentinan (LNT)-functionalized SeNPs (Selene) and treated tumor cells Ehrlich ascites cancer and OVCAR-3 malignant ascites. Their effect on mitochondrial function was studied using KEGG and GO analysis. However, the mitochondrial membrane potential decreased and the reactive oxygen species (ROS) level increased after Selene treatment. Further testing revealed that Selene increases the expression of caspase-3, a key apoptotic enzyme, reduces the level of ATP, and is better than SeNPs. The dynamic mechanism of their entry into cells was also investigated. Selene can specifically target the mitochondria of tumor cells through the TLR4/TRAF3/MFN1 pathway. This targeting not only induces apoptosis in tumor cells but also inhibits the secretion of inflammatory cytokines in ascites [66]. Therefore, the primary anti-tumor mechanism of SeNPs is to induce cellular oxidative stress and mitochondrial dysfunction, thereby promoting apoptosis. The superior anti-tumor activity of SeNPs is not a singular event but a cascade of interconnected mechanisms, primarily initiated by their unique physicochemical properties. Their high surface-area-to-volume ratio and modifiable surface chemistry are pivotal for cellular uptake and specific targeting. Additionally, SeNPs can selectively inhibit specific oncogenic signaling pathways to suppress tumor growth. Androgen and the androgen receptor play key roles in the treatment of prostate cancer. The current strategy used in treating prostate cancer is to block the synthesis of androgen and the androgen receptor, and Kong et al. have found that SeNPs partially inhibit the growth of prostate LNCaP cancer cells through caspase-mediated apoptosis. SeNPs inhibit the transcriptional activity of the androgen receptor by downregulating the androgen receptor mRNA and the protein expression. In addition, SeNPs achieve an anti-cancer effect by activating the Akt/Mdm 2 pathway to degrade the androgen receptor [67]. In addition, SeNPs can also effectively block the proliferation and metastasis of tumor cells by inhibiting angiogenesis in vivo. Wang et al. assessed the impacts of varying concentrations of *P. lactiflora* polysaccharide-based SeNPs (PLP-SeNPs) on blood vessel formation in a transgenic zebrafish model, and found that the intersegmental vasculature (isv) of zebrafish embryos was significantly broken or absent and the length of the isv was reduced under the effect of different concentrations of PLP-SeNPs [68] (indicated by red arrows). Immune cell therapy is one of the most important aspects of tumor immunotherapy [69]. Additionally, SeNPs possess certain immunomodulatory functions. Some scholars have found that the effects of SeNPs are similar to that of nano-enzymes, which can accelerate the tubulin acetylation of T cells and enhance the toxicity of T cells [70]. Most of the anti-tumor mechanisms of SeNPs are summarized in Table 1. Despite these promising mechanisms, significant challenges remain. A critical gap lies in the frequent disparity between robust in vitro findings and more complex in vivo outcomes, necessitating further translational studies. Moreover, the potential for off-target toxicity, where SeNPs might inadvertently affect healthy cells through similar oxidative stress mechanisms, requires thorough investigation to ensure therapeutic safety and specificity. Future research should focus on engineering SeNPs with superior targeting capabilities to maximize anti-cancer efficacy while minimizing adverse effects.

**Figure 5:**
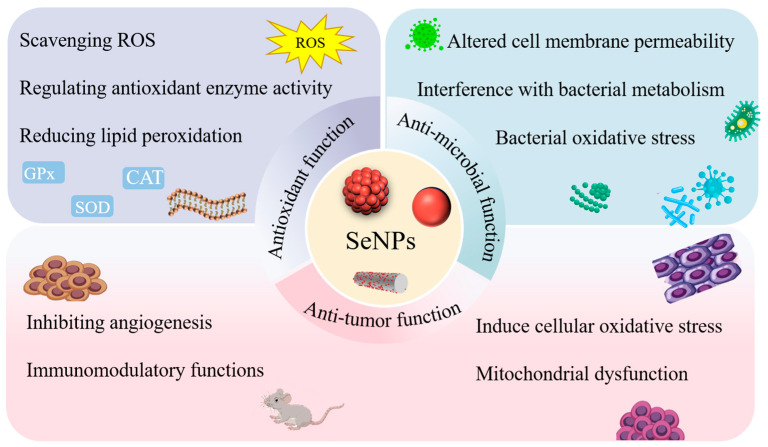
The anti-tumor, antioxidant and antibacterial mechanisms of SeNPs (Some schematic elements were downloaded from “Vecteezy” website; the elements downloaded from this website are free).

### 3.2. The Mechanisms of Antioxidant Function

**Figure 6:**
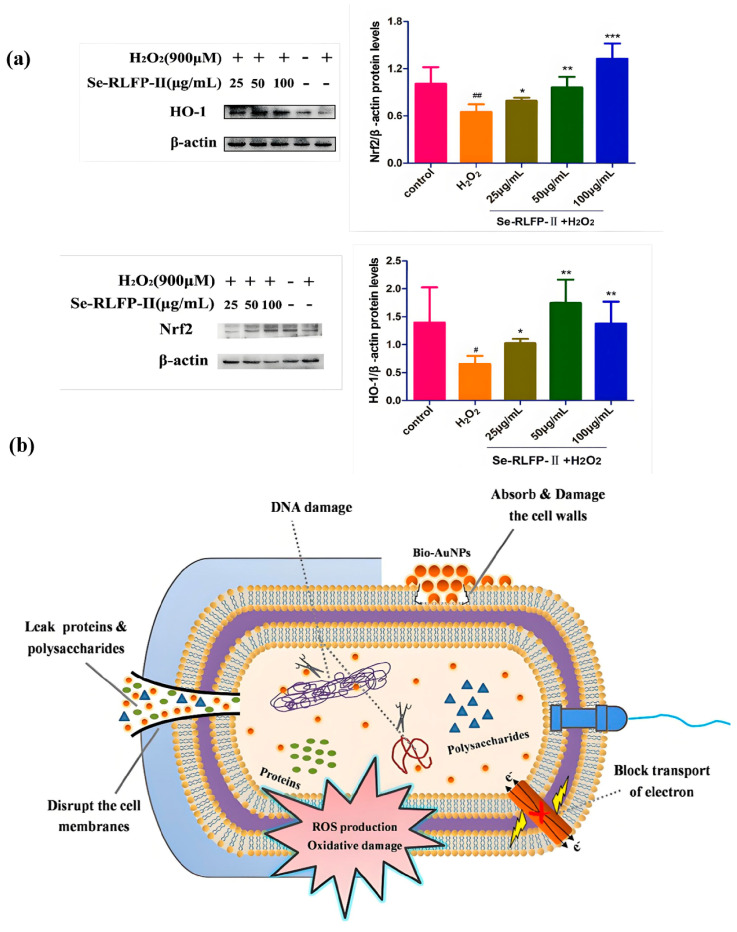
Typical antioxidant and anti-microbial functions of SeNPs, respectively. (**a**) SeNPs activate the Nrf2/HO-1 signaling pathway and effectively improve antioxidant activity in neuronal cells (Compared with the control group, ^#^ *p* < 0.05, ^##^ *p* < 0.01; compared with the H_2_O_2_ treated cell group, * *p* < 0.05, ** *p* < 0.01, *** *p* < 0.001), adapted with permission from the copyright 2022, Elsevier [84]. (**b**) SeNPs produce bacteriostatic effects by altering the permeability of cell membranes, adapted with permission from the copyright 2020, Elsevier [95].

Zhang et al. study the neuroprotective effects of *Lycium barbarum* polysaccharidesgreen tea—SeNPs on cell death induced by oxidative stress using the MTT assay. The survival rate of cells treated with H_2_O_2_ is 90%, indicating that SeNPs can protect cells from H_2_O_2_-induced damage, and, thus, they have good levels of antioxidant activity [83]. Liu et al. extract two selenium polysaccharides (Se RLFPs) from *Rosa laevigata* Michx fruits and show the high neuroprotective activity in the human neuroblastoma SH-SY5Y cell injury model induced by H_2_O_2_. They significantly increase total antioxidant capacity and superoxide dismutase activities, while significantly inhibiting malondialdehyde (MDA) content and exerting antioxidant capacity. The Nrf2/HO-1 pathway is a well-known signaling cascade involved in antioxidant stress responses. Western blot analysis reveals that Se-RLFP-II significantly upregulates the expression of Nrf2 and HO-1 in SH-SY5Y cells. Consequently, it can effectively mitigate neuronal cell damage induced by H_2_O_2_ through the activation of the Nrf2/HO-1 pathway, thereby enhancing antioxidant activity [84] (as shown in Figure 6a). SeNPs with an appropriate particle size can enhance the ability to penetrate tissues and cells, effectively inhibit the accumulation of ROS, reduce the cytotoxicity of Se, protect glutathione peroxidase (GPx) activity, and prevent the D-galactose-induced accumulation of lipofuscin [85]. Selenium is an essential nutrient for fattening pigs. During the process of feeding the pigs with a basic diet supplemented with glycine selenium nanoparticles (G-SeNPs), the total antioxidant capacity, glutathione peroxidase, superoxide dismutase, and catalase activities in pig serum increase linearly with the concentration of G-SeNPs. As well as their enhanced antioxidant capacity, the growth performance and meat quality of pigs are not adversely affected [86]. Extensive studies have demonstrated that the antioxidant effects of SeNPs in organisms are primarily attributed to its functions of scavenging ROS, regulating antioxidant enzyme activity, and reducing lipid peroxidation. These functions of SeNPs are attributed to their unique nano-size properties and controlled release characteristics. Most of the antioxidant mechanisms of SeNPs have been listed in Table 2.

### 3.3. The Mechanisms of Anti-Microbial Function

The integrity of cell membranes is a key factor in bacterial growth. The cell membrane not only maintains the stability of the cell energy and the material metabolism environment but also regulates and selects the substances entering and leaving the cell. It is believed that the functional mechanism of SeNPs is to attach particles to the bacterial surface and release selenium ions into bacterial cells, causing oxidative stress, the inhibition of protein synthesis or DNA mutations [93]. Xu et al. investigated the integrity of the cell membrane in *Pseudomonas fluorescens* ATCC 13525 by measuring the leakage of proteins and nucleic acids. The results indicated that SeNPs can compromise the integrity of the cell membrane, thereby exerting antibacterial effects. Following treatment with SeNPs, the intracellular concentration of adenosine triphosphate (ATP) and antioxidant enzyme activity were found to decrease, while the levels of ROS and MDA increased. This led to intensified lipid peroxidation and ultimately resulted in cell death [94]. Zhang et al. used *Provincia* sp. DCX for the biosynthesis of SeNPs; most of the gram-negative bacterium were locally killed after 12 h of treatment with 500 mg/L biological SeNPs in the antibacterial experiment. Except for *Bacillus subtilis*, the killing effect of gram-positive bacterium is better. In the experiment measuring the leakage of proteins and polysaccharides, an increased amount of these substances was found outside the cell. This indicates that the addition of biological SeNPs alters the permeability of the cell membrane, thereby exerting antibacterial effects. The experiment also detected the content of ROS, and the antibacterial mechanism of SeNPs was related to the oxidative damage caused by the increase in ROS [95] (as shown in Figure 6b). The increase in biosynthetic SeNPs concentration may lead to the increase in ROS, and may have strong antibacterial activities, as the generated ROS exert pressure on the bacterial cell membrane, causing the leakage of cytoplasmic material in bacterial cells, so that the cell cannot survive [96]. Shin et al. synthesized SeNPs using *Cirsium setidens* extracts and ascorbic acid, which are non-toxic to the normal fibroblast cell line NIH3T3 but cytotoxic to the human lung cancer cell line A549. They may inhibit the proliferation of A549 cell lines by damaging the nucleus and mitochondrial membrane [97]. SeNPs can also inhibit the activity of key enzymes involved in microbial metabolism, thereby disrupting bacterial metabolic processes, and suppressing microbial growth and proliferation [98]. Consequently, the antibacterial mechanism of SeNPs results from the synergistic effects of bacterial oxidative stress, altered cell membrane permeability, and the interference with bacterial metabolism. This multi-target mode of action reduces the likelihood of bacteria developing resistance, making SeNPs a promising candidate for combating drug-resistant pathogens.

## 4. Applications in the Biological Field

### 4.1. Application in Packaging Materials

**Figure 7:**
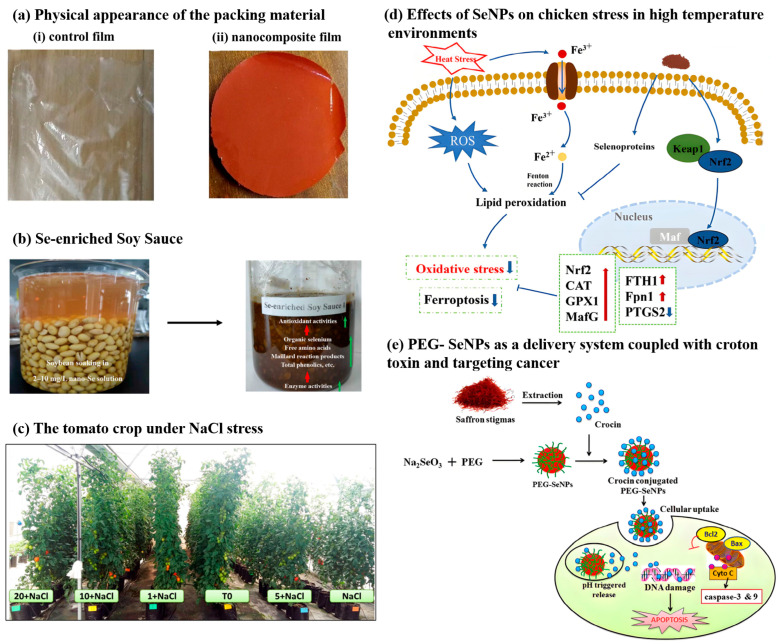
(**a**) The applications of SeNPs as the typical packaging material, adapted with permission from the copyright 2022, Wiley [100]. (**b**) The applications of SeNPs in selenium-rich food, adapted with permission from the copyright 2022, Polish Society for Horticultural Science [105]. (**c**) The applications of SeNPs in fertilizers, adapted with permission from the copyright 2019, MDPI [114]. (**d**) The applications of SeNPs in animal feed, adapted with permission from the copyright 2023, MDPI [120]. (**e**) The applications of SeNPs as drug carriers, adapted with permission from the copyright 2016, RSC [78].

So far, a variety of inorganic and metal nanoparticles have been utilized in the development of active food packaging materials. These nanoparticles help extend the shelf life of food products by reducing their reliance on chemical preservatives and accelerating the reactions that inhibit microbial growth [99]. Possessing excellent antibacterial and antioxidant activities, SeNPs can be incorporated into food packaging materials to harness their multifunctional properties for enhanced food preservation. The potato SeNPs’ composite films (SeNPs were used at 1 mg/mL), prepared by Ndwandwe et al., have strong moisture resistance properties and are suitable for food packaging (as shown in Figure 7a). The addition of SeNPs improves the antibacterial properties of the films against the tested microorganisms (*Salmonella typhimurium*, *Escherichia coli*, and *Bacillus cereus*). Although SeNP composite films exhibit low fluidity and are thus applicable for packaging acidic, hydrophilic, and high-fat foods, the potential health risks associated with chronic dietary exposure remain elusive due to the lack of comprehensive toxicological data [100]. Active packaging incorporating (SeNPs) effectively prevents food oxidation and extends shelf life. Vera et al. conducted studies on the oxidation of hazelnuts, walnuts, and potato chips, demonstrating that SeNPs possess potent free radical scavenging properties. The final concentration of SeNPs was approximately 100 mg/kg [101].

Beyond bioactivity, the incorporation of SeNPs improves the physical and protective properties of the packaging matrix itself. Jamróz et al. used furfural (FUR) and gelatin (GEL) aqueous solutions as raw materials. Different concentrations of nanoparticles (Se-AgNPs) were added to prepare composite films. As the concentration of Se-AgNPs increased, the elasticity and elongation at break of the material were altered. This study also discussed the physical properties, optical properties, tensile behavior, water vapor transition rate, thermal properties, anti-microbial properties, and storage time of nanocomposite films. The addition of Se-AgNPs to FUR/GEL films enhanced the UV light barrier properties, mechanical strength, and the thermal stability of the resulting nanocomposite. Storage studies demonstrate that incorporating Se-AgNPs (approximately 25–75 mg/mL) into FUR/GEL films can effectively extend the shelf life of kiwis, suggesting their potential application in food preservation. However, it still needs further exploration to determine whether it can be used as a safe packaging material [102,103]. Some studies have synthesized SeNPs using the extract of *Annona chinensis* leaves as a reducing agent, and have prepared chitosan-*Andrographis paniculata* extract-SeNPs (CS-APE-Se). The prepared films possess good levels of biodegradability after 30 days of burial and have certain antibacterial properties. After using the films of CS-APE-Se (approximately 0.1–0.4 mg/mL), the preservation performance of strawberries is improved, substantially decreasing spoilage and extending the shelf life to 10 days [104].

### 4.2. Application in Selenium-Rich Functional Foods

SeNPs also have an impact on the nutritional quality of foods. Bean sprouts are very common in daily life. A comparative study was conducted to assess the effects of SeNPs and selenite on the selenium content and nutritional quality of bean sprouts (as shown in Figure 7b). The results have indicated that both SeNPs and selenite exert significant effects on the growth of soybean sprouts. The carotenoid content in soybean sprouts is significantly elevated with increasing dosages of SeNPs (0.789–7.896 mg/L). While selenite treatments also enhance carotenoid levels, this effect is observed only at specific concentrations, of 1.73 mg/L and 13.84 mg/L. Critically, SeNPs reduced MDA accumulation while enhancing glutathione (GSH) levels, which can be attributed to their antioxidant surface properties and sustained release capacity, thereby reinforcing the cellular defense system. Overall, SeNPs appear to be a more appropriate selenium source for the development of selenium-enriched soybean sprouts compared to selenite [105]. Moreover, the biocompatibility and high bioavailability of SeNPs make them ideal vehicles for selenium fortification in food matrices. Soy sauce serves as an optimal delivery vehicle for selenium supplementation in human diets. During the fermentation process of soy sauce, microbial transformations facilitate the formation of organic selenomethionine and selenocysteine. A highly effective method for producing selenium-enriched soy sauce involves soaking soybeans in a solution of SeNPs. When the concentration of selenium is maintained at an appropriate level (6 mg/L SeNPs), it significantly enhances the total selenium and organic selenium content in the soy sauce [106].

### 4.3. Application in the Fertilizer Field

Traditional fertilizers and pesticides face the problems of low utilization efficiency and potential harm to non-target organisms. However, SeNPs, with their unique nanoscale effect, controlled release property, and low biotoxicity, have attracted widespread attention and have shown great potential in agricultural applications [19,107,108]. Driving nutrient cycling in the soil, rhizosphere microorganisms are essential drivers of productivity and health in agricultural ecosystems. Plant growth and the cycling of selenium depend on a stable and robust soil microbial community. SeNPs enter microorganisms at a slower rate, with lower toxicity and higher safety levels. SeNPs are more stable in soil than SeO_3_^2−^ [109]. Selenite and selenate have been widely used in foliar selenium fertilizers in the production of selenium-rich rice. Lidon et al. demonstrated that selenium biofortification, through the application of Na_2_SeO_3_ and sodium selenate, can enhance the contents of lipids, sugars, and proteins in rice. However, its selenium application rate is relatively high (120–300 g/ha Se), and the safety assessment is not sufficiently comprehensive [110]. Compared with Na_2_SeO_3_ and sodium selenate, SeNPs are more equivalent to a slow-release fertilizer and effect crops for a longer duration. Appropriate concentrations of SeNPs (6.4 g/ha) can promote the formation of various volatile organic compounds, improve rice aroma, and significantly affect the characteristics of rice grains [111]. When applying selenium to the leaf surface, excessive spray concentration (76.87 g/ha Se) may cause damage to crop leaves [112]. Studies have shown that SeNPs (20–40 mg/L) can improve the antioxidant defense system of crops and enhance their ability to tolerate stress [113]. Salinity impacts crop productivity, affects the absorption of other nutrients, and is directly harmful to crops. Morales-Espinoza et al. applied SeNPs (20 mg/L) to tomatoes under salt stress and found that the yield of the plants increases, probably as a result of the increased number of photosynthetic pigments in the leaves (as shown in Figure 7c). The increase in antioxidant compounds in the fruit, such as lycopene and β-carotene, improved the tolerance of tomato plants to salt stress [114]. At present, research on selenium in radish has also made certain progress. Applying SeNPs (90 mg/L) not only promotes the growth of radishes but also transports nutrients underground. Exogenous supplementation of SeNPs can considerably improve yield and quality traits [115]. However, despite the notable advantages of SeNPs in agricultural applications, their environmental behavior and biosafety require systematic evaluation.

### 4.4. Application in Animal Feed

SeNPs have been extensively used in poultry nutrition because of their excellent antioxidant properties. Several enzymes in the antioxidant system include superoxide dismutase, glutathione peroxidase, and catalase. Saleh et al. added SeNPs (0.5 mg/kg) to feed broilers and found that adding SeNPs can increase the expression of glutathione peroxidase and superoxide dismutase mRNA in broiler muscles, improve the growth performance of broilers, and raise the selenium content and antioxidant capacity in muscles [116]. Leveraging these superior antioxidant properties, SeNPs hold significant promise in mitigating heat stress in poultry. Poultry animals are very sensitive to temperature and are prone to heat stress. Oxidative stress caused by heat stress can significantly reduce the body’s antioxidant defense ability, induce the inflammatory response, and reduce the food intake and growth rate of animals [117]. Sheiha et al. compared the effects of biosynthesized SeNPs and chemically synthesized SeNPs on the growth, oxidation, and inflammation of rabbits under heat stress conditions. Bio-synthesized SeNPs (25–50 mg/kg) significantly improved antioxidant indicators and inflammatory factor response. Furthermore, the limited efficacy of chemically synthesized SeNPs at the high dosage (50 mg/kg) suggested their higher potential toxicity [118]. Debata et al. studied the effects of SeNPs on chicken stress under excessive temperature environment. They found that, after stress, chicken productivity decreases, and SeNPs (0.15–0.3 mg/kg), as antioxidants, can promote the metabolism, and the enzymatic and biochemical reactions in poultry [119,120] (as shown in Figure 7d). Nevertheless, despite the marked benefits, the large-scale application of SeNPs in poultry farming faces challenges, including the long-term safety profile, tissue residue dynamics, and the lack of clear regulatory standards, which are critical issues that must be addressed before widespread industrialization.

### 4.5. Application as Drug Carriers

In recent years, SeNPs have emerged as highly promising drug delivery carriers due to their excellent biocompatibility, low toxicity, and high loading capacity [121]. Mai et al. developed an astragalus polysaccharide-functionalized SeNPs system co-loaded with tanshinone IIA and tetramethylpyrazine (TMC-Se-M-NLCs). This system successfully integrates anti-ferroptosis with anti-inflammatory regulation, demonstrating synergistic therapeutic efficacy in the treatment of spinal cord injuries (TMC-Se-M-NLCs administration concentration was approximately 1 mg/kg), thereby highlighting the potential of SeNPs as a platform for modernized combination therapy in traditional Chinese medicine [122]. Although new drug delivery technologies are constantly being developed, their effective delivery to potential diseases within macrophages still needs to be validated [123]. SeNPs serve as versatile drug carriers, capable of co-delivering multiple active ingredients to achieve enhanced therapeutic efficacy. Studies have shown that the mannosylation of non-carriers can increase the uptake of macrophages in target tissues or organs [124]. Rojekar et al. used mannose as a lipid carrier, loaded with SeNPs and anti-HIV drugs to form a nanostructured lipid carrier, and delivered it to the HIV virus site (SeNPs administration concentration was approximately 0.022 mg/kg). The effect of the drug was evaluated through in vitro experiments and in vivo biological distribution studies. Compared with conventional drugs, the drug had a higher therapeutic effect, a significantly improved therapeutic effect, and reduced the potential of all major organs in oxidative stress [125]. Liu et al. developed lentinan-stabilized SeNPs co-loaded with 2,3,5,6-tetramethylpyrazine and ginsenoside Rg1 for spinal cord injury treatment, which not only exhibited remarkable antioxidant properties but also alleviated microglial pyroptosis via the NLRP3/caspase-1 pathway and promoted axonal regeneration, leading to significantly improved motor function recovery in model mice (SeNPs administration concentration was approximately 0.345 mg/kg) [126]. SeNPs, as a biological nanomaterial, have created new prospects for cancer treatment. Selenium may be administered alongside conventional chemotherapy or established anti-cancer drugs as part of a treatment regimen. Liu et al. utilized the anti-metabolic drug 5-fluorouracil, which was used to treat tumors via binding with SeNPs (SFU-SeNPs). The modification of 5-fluorouracil significantly enhanced the uptake of SeNPs by intracellular phagocytosis and had great selectivity between normal and cancer cells, making it more effective in cancer cells. Using SeNPs as a carrier for anti-cancer drugs to achieve synergistic anti-cancer effects provides candidate drugs for screening anti-cancer drugs. The lethal dose (LD_50_) of SFU-SeNPs in mice was 108.6 mg Se/kg [31]. Mary et al. found that PEG-SeNPs can act as a drug delivery system, coupling to crocin and targeting cancer, making drug delivery more efficient and being a highly efficient way to achieve the synergistic treatment of lung cancer (as shown in Figure 7e). PEG-SeNPs loaded with crocin have shown very high efficacy against lung cancer cells. Their 48 h IC_50_ value only requires a selenium concentration of 0.62 µg/mL, which is much lower than the concentration that is significantly toxic to normal cells (which is still shown as safe up to 3.16 µg/mL in the experiment), and their anti-cancer effect is much higher than that of crocin alone [78]. Metabolic diseases, such as type II diabetes and cardiovascular diseases, pose major challenges to global health problems, and the incidence rate is increasing.

Studies have shown that many nano drug delivery systems composed of liposomes, nanoparticles, hydrogel nanocomposites, and plant extracts have effectively reduced insulin resistance, oxidative stress, and chronic inflammation because of the capacity of plant extracts to modulate the pathways associated with metabolic syndrome. Therefore, the comprehensive application of SeNPs and plant extracts can provide a reference for clinical applications [127]. SeNPs also demonstrate excellent antioxidant activity and biocompatibility in drug delivery systems. For instance, Bi et al. developed a self-healing hydrogel, based on chitosan/cellulose nanofibers/SeNPs, as a carrier for adipose-derived mesenchymal stem cells in diabetic wound therapy. This hydrogel is not only injectable and self-healing but also protects the stem cells by scavenging reactive oxygen species, thereby significantly accelerating wound healing, collagen deposition, and angiogenesis. The optimal balance between the antioxidant protective effect of SeNPs and the avoidance of cytotoxicity is 0.395 µg/mL [128]. Hence, the potential toxicity of SeNPs is dose-dependent, and the translation of SeNPs-based drug delivery systems to clinical applications still faces safety and regulatory challenges. Future research must prioritize rigorous in vivo toxicological studies and develop strategies for their targeted delivery and controllable biodegradation, to ensure their safety in biomedical applications.

## 5. Conclusions

In summary, this review provides a comprehensive analysis of the synthesis, functional mechanisms, and applications of SeNPs. Its uniqueness lies in the focused elucidation of the structure–activity relationship between SeNPs synthesis mechanisms and biological functions. A key and clear finding is that biologically derived SeNPs exhibit excellent anti-cancer selectivity, showing stronger cytotoxicity toward cancer cells while having minimal effects on normal cells, laying the foundation for their use as therapeutic agents. A growing body of research has focused on developing eco-friendly and sustainable biosynthetic methods. Additionally, this paper details the antioxidant and antibacterial mechanisms of SeNPs, which support their applications in the fields of food packaging materials, functional foods, animal feeds, plant fertilizers, and biomedicine. Currently, SeNPs are approaching having a practical application in food packaging, functional foods, animal feed, and plant fertilizers; however, large-scale production processes, long-term safety, and regulatory approvals still require in-depth exploration. Their specific efficacy, stability, and safety in the human body await further validation through clinical studies. On the other hand, the application of SeNPs in the biomedical field remains in the research stage. Current research focuses on precise functional design and clinical translation, which necessitate complex clinical trials and rigorous regulatory review.

## 6. Future Perspectives

While SeNPs exhibit a markedly improved safety profile compared to their inorganic and organic counterparts, a thorough understanding of their toxicology is paramount to guiding their safe application. Research has found that the acute oral LD_50_ of *Sargassum fusiforme* polysaccharide-modified SeNPs is 88.76 mg Se/kg, which is substantially higher than the 28-day repeated dose no-observed-adverse-effect level (NOAEL) of Na_2_SeO_3_ in mice (4.43 mg Se/kg/day). This indicates that SeNPs effectively reduce the acute toxicity of selenium [129]. The toxicity of SeNPs is fundamentally dose-dependent and influenced by key physicochemical properties, such as size, surface charge, and coating agents. Stepankova et al. systematically evaluated the toxicological properties of SeNPs with different morphologies and aspect ratios. The study revealed that Se nanorods with higher aspect-ratio (AR) value (SeNR-2, AR ≈ 22.3) exhibited stronger cytotoxicity against both healthy cells and cancer cells in vitro, and induced significant kidney damage in mice after intravenous injection. In contrast, low-aspect-ratio nanorods (SeNR-1, AR ≈ 11.5) demonstrated optimal biocompatibility with an in vitro IC_50_ exceeding 100 µg/mL [9]. In a comparative assessment of bioinspired nanomaterials using a zebrafish embryo model, Krishnaraj et al. reported that SeNPs exhibited high toxicity, causing embryo coagulation at a concentration as low as 1.56 μg/mL. In contrast, chitosan-based SeNPs significantly mitigated this toxicity, with no observed coagulation at concentrations up to 25 μg/mL, highlighting the role of selecting appropriate biocompatible matrices in reducing the toxicity of nanomaterials [130]. Therefore, the biological safety of SeNPs not only depends on their core components, but is also profoundly influenced by their physical form (such as aspect ratio) and surface chemistry (such as polysaccharide and chitosan modification). Therefore, through reasonable material design, their biological safety can be significantly enhanced while retaining their functional activity, laying the foundation for future biomedical applications.

Despite notable advancements, the translation of SeNPs into practical use still encounters several distinct challenges that will guide future research efforts. Firstly, emphasis should be placed on ensuring reproducibility and scalability by accurately controlling the size and shape of SeNPs through fine-tuning synthesis parameters. It is crucial to develop synthesis methods that are efficient, controllable, and environmentally friendly, to guarantee consistency between batches and long-term stability. Moreover, creating standardized protocols for characterizing SeNPs is necessary to allow for meaningful comparisons of effectiveness to be drawn across various studies. Lastly, enhancing toxicological studies and preclinical safety evaluations of SeNPs should be prioritized. Our aim is to provide a clear framework for assessing the technological maturity of SeNPs and guiding future research priorities. Although SeNPs exhibit much lower acute toxicity compared to inorganic selenium, smaller particles with unmodified surfaces are more prone to entering the human body due to their size and surface properties, which may present potential risks. Consequently, future research should focus on the development of more efficient precise synthesis methods and stabilizers to improve the bioactivity and stability of SeNPs, enabling the application of SeNPs in more fields.

## Figures and Tables

**Table 1:** Recent works on SeNPs with typical functional mechanisms as the potential anti-cancer agents.

Synthesis Methods	Synthesis Condition	Size (nm)	Shape	Cancer/ Normal Cell Lines	Anticarcinogenic Action	Reference
Biosynthesis method	*Streptomyces parvulus* MAR4, Na_2_SeO_4_, chitosan	~476	Spherical	HepG2, Caki-1 (HTB-46)/WI-38	Nanoconjugates with a large number of positive charges can interact electrostatically with negatively charged cell membranes, leading to cellular damage.	[71]
Biosynthesis method	*Bacillus licheniformis* JS2, Na_2_SeO_3_	~110	Spherical	PC-3	ROS generation and mitochondrial damage, the activation of TNF and IRF1 genes.	[72]
Biosynthesis method	*Kaempferia parviflora* (black ginger) root extract, Na_2_SeO_3_	~214	Spherical	AGS cells/HaCaT	PI3K/Akt/mTOR pathway	[73]
Chemical method	Vc, Na_2_SeO_3_, hyaluronic acid, Doxorubicin	~78	Spherical	HeLa	The induction of apoptosis in HeLa cells via the Bcl-2 signaling pathway. This process involves downregulating the expression of the Ki67 protein, and the caspase-3 apoptosis-related signaling pathway is activated to further promote cell apoptosis.	[74]
Chemical method	Glucose, Na_2_SeO_3_	~280	Spherical	HepG2, MCF7, A549, Neuro-2a, A375/HK-2	Caspase-8 (Fas/TNF—mediated), caspase-9 (mitochondria mediated) and caspase-3 are dose-dependent, and activated by SeNPs, leading to apoptosis of HepG2 cells. The decline of mitochondrial membrane potential (ΔΨm).	[75]
Biosynthesis method	Vc, Na_2_SeO_3_, *Gracilaria lemaneiformis* polysaccharide	~100	Spherical	U87 and C6	The dose-dependent activation of caspase-3, caspase-8, and caspase-9 indicates that both the death receptor-mediated and mitochondria-mediated pathways play roles in SeNPs-induced apoptosis. MAPKs and AKT signal pathways.	[76]
Biosynthesis method	*Trifolium cherleri* aerial parts, Na_2_SeO_3_, niosome	~178	Spherical	MCF-7, T47D and MDAMB231/HFF	Niosome-loaded SeNPs are capable of upregulating the expression levels of apoptosis-related genes, including Bax, caspase-3, and caspase-9. Conversely, the expression of the antiapoptotic gene Bcl-2 is downregulated.	[77]
Chemical method	PEG200, Na_2_SeO_3_, crocin	~31	Spherical	A549	SeNPs demonstrated increased cytotoxic effects on A549 cells by inducing apoptosis through a mitochondria-mediated pathway. Additionally, these nanoparticles effectively inhibited tumor growth in an in vivo nude mice model.	[78]
Chemical method	Vc, Na_2_SeO_3,_ transferrin, chitosan	~130	Spherical	A375, HepG2 and MCF-7/HUVEC	The internalization of SeNPs induces excessive production of intracellular ROS, thereby activating the p53 and MAPK signaling pathways, which subsequently promote cell apoptosis. In in vivo studies using a nude mice model, SeNPs have been shown to significantly inhibit tumor growth by inducing apoptosis mediated through the p53 pathway.	[79]
Biosynthesis method	*Morinda officinalis* polysaccharide, Vc, Na_2_SeO_3_	~67	Spherical	HepG2, MCF-7, AGS, PC9 and HCT8	Inducing cell circle G_0_/G_1_ phase arrest.	[80]
Chemical method	*Grateloupia livida* polysaccharides, Vc, Na_2_SeO_3_	~116	Spherical	A549	S phase cell cycle arrest regulated the anti-proliferation effect of SeNPs. The decrease in mitochondrial membrane potential induced A549 cell apoptosis.	[81]
Biosynthesis method	Protamine sulfate, Vc, Na_2_SeO_3_	~130	Spherical	HepG2	The proliferation of HepG2 cells was inhibited by blocking S phase, up-regulating ROS level, and inducing apoptosis.	[82]

**Table 2:** Recent works on SeNPs with typical functional mechanisms as the potential antioxidant agents.

Synthesis Methods	Synthesis Condition	Size (nm)	Shape	Antioxidant Action	Reference
Biosynthesis method	*Mori fructus,* polysaccharides, Na_2_SeO_3_, Vc,	~80	Spherical	Reducing ROS levels and enhancing antioxidant enzyme activity.	[87]
Biosynthesis method	*Moringa oleifera Lam*, Na_2_SeO_3_, Vc,	~166	Spherical	SeNPs could upregulate the activity of antioxidant enzymes in HepG2 cells, suppress H_2_O_2_-induced ROS generation, and alleviate oxidative damage to cell membranes.	[88]
Chemical method	Hyaluronic acid, Na_2_SeO_3_, Vc	~82	Spherical	The direct elimination of ROS and effective alleviation of oxidative stress through selenite-induced selenoprotein synthesis have been accomplished, along with optimization of mitochondrial-related oxidative phosphorylation pathways.	[13]
Chemical method	Chlorogenic acid, human serum albumin, Na_2_SeO_3_, Vc	~35	Spherical	SeNPs have scavenged multiple ROS and downregulated the expression of Mapk8ip1 (MAPK pathway) and Itga2b (PI3K-Akt pathway).	[89]
Chemical method	Polyvinyl alcohol or chitosan, Na_2_SeO_3_, Vc	~83 ~63	Spherical	SeNPs have catalyzed NO generation and consumed ROS.	[90]
Chemical method	Selenium dioxide, chitosan, Vc	~50	Spherical	The direct scavenging of ROS and upregulation of antioxidant enzyme activities have been achieved, thereby alleviating oxidative damage caused by arsenic stress.	[91]
Chemical method	Chitosan, Na_2_SeO_3_, Vc	~95	Spherical	Enhanced selenium retention and glutathione peroxidase levels have been achieved, along with reduced lipid peroxidation.	[92]

## Data Availability

No new data were created or analyzed in this study. Data sharing is not applicable.

## Supplementary Materials

The following supporting information can be downloaded at: https://www.mdpi.com/article/10.3390/foods14213640/s1, The various synthesis method is detailed in Table S1, Table S2, Table S3, Table S4.
