# Supplementary material for: Recent Advances Towards Selenium Nanoparticles: Synthetic Methods, Functional Mechanisms, and Biological Applications
Source: Foods. 2025 Oct 24;14(21):3640. doi: 10.3390/foods14213640 (PMC12607590; doi:10.3390/foods14213640)
Supplement: Supplementary file 1 [file foods-14-03640-s001.zip › foods-3925752-supplementary.pdf]

foods

Supplementary Materials

Recent advances towards selenium nanoparticles: Synthetic methods, functional mechanisms, and biological applications

Lulu Geng [1,2], Linling Li [2,*], Xuening Sun [1,2], Shuiyuan Cheng [1,2] and Jiangling He [1,*]

1 National R&D Center for Se-rich Agricultural Products Processing, Wuhan Polytechnic University, Wuhan, 430023, PR China;
2 School of Modern Industry for Selenium Science and Engineering, Wuhan Polytechnic University, Wuhan, 430023, PR China;
* Correspondence: lilinling1437@126.com (L. Li); hejiangling@whpu.edu.cn (J. He)

Table S1. The synthesis conditions and characteristics of SeNPs by physical methods.

Synthesis conditions	Size (nm)	Shape	Zeta potential (mV)	References
Selenium source: A high-purity selenium target Laser parameters: 24 fs, 800 nm, 1–7 nJ, 80 MHz repetition rate<br>Target: High-purity selenium<br>Solvents: De-ionized water, ethanol, methanol, isopropanol, n-hexane<br>Ablation time: 1 to 45 mins	5-200	Quasi-spherical or ribbon-like structures	/	[1]
Selenium source: Bulk Se pellets<br>Laser: Q-switched Nd:YAG, 1064 nm, 70-200 ns, 3000 Hz<br>Solvent: Deionized water	~43	Spherical	-66	[2]
Selenium source: H_2SeO_3<br>Reducing agent: $N_2H_4{\cdot}H_2O$<br>Ultrasound: 20-300 W, 25 kHz	~600<br>~300	Nanorods, nanotubes nanospheres	/	[3]
Selenium source: Na_2SeO_3<br>Reducing/Capping agent: Theobroma cacao L. bean shell extract<br>Microwave irradiation: 788.6 W for 15.6 min	~3	Spherical	-28.6	[4]
Selenium source: Na_2SeO_3<br>Reducing agent: L-asparagine<br>Capping agent: PVP<br>Microwave-assisted: 5-25min	~50<br>~130	Nanoball, nanotube, multi-armed nanorod	/	[5]

Table S2. The synthesis conditions and characteristics of SeNPs by chemical method

Synthesis conditions	Size (nm)	Shape	Zeta potential (mV)	References
Selenium source: Na_2SeO_3<br>Reducing agent: Vc<br>Stabilizers/Capping agent: PVA or chitosan.	66	spherical	/	[6]
Selenium aource: Na_2SeO_3<br>Reducing agent: Glucose<br>Stabilizing agent: Glycerin and 3-mercaptopropionic acid	~5	Nanorods	/	[7]
Selenium source: $SeCl_4$<br>Reducing agent: Thiourea<br>Stabilizing agent: Chitosan	50	Spherical	/	[8]
Selenium source: H_2SeO_3<br>Reducing agent: Vc<br>Stabilizing agent: Chitosan or sodium carboxymethyl cellulose	~42<br>~43	Spherical	/	[9]
Selenium source: H_2SeO_3<br>Reducing agent: Vc<br>Stabilizing agent: Chitosan, κ-carrageenan, gum arabic	~387<br>~203<br>~132	Spherical<br>Irregular<br>Irregular	~+30<br>~-30	[10]

Table S3. The synthesis conditions and characteristics of SeNPs by biosynthesis method.

Synthesis conditions	Size (nm)	Shape	Zeta potential (mV)	References
Selenium source: Na_2SeO_3<br>Reducing agent: *Metasolibacillus* sp. ES129, *Oceanobacillus* sp. ES111	~200<br>~100	Spherical	/	[11]
Selenium source: Na_2SeO_3<br>Reducing agent: *Saccharomyces cerevisiae*	~75	Spherical	-10	[12]
Selenium source: Na_2SeO_3<br>Reducing agent: Ascorbic acid<br>Stabilizing agent: *Morchella sextelata* polysaccharide	~72	Spherical	/	[13]
Selenium source: Na_2SeO_3<br>Reducing/Stabilizing agent: Bacillus subtilis T5 and metabolites	~167	Spherical	-31	[14]
Selenium source: Na_2SeO_3<br>Reducing/Stabilizing agent : *Lactobacillus paralimentarius* JZ07 and metabolites	~200	Spherical	/	[15]

Selenium source: Na_2SeO_3<br>Reducing/Stabilizing agent: *Aspergillus flavus* and metabolites	~50	Spherical	/	[16]
Selenium source: Na_2SeO_3<br>Reducing/Stabilizing agent: *Tetrahymena thermophila* cells	~200	Spherical/ irregular		[17]
Selenium source: Na_2SeO_3<br>Reducing/Stabilizing agent: Pomegranate Peel Extract	~9	Spherical	-31.4	[18]
Selenium source: Na_2SeO_3<br>Reducing/Stabilizing agent: Citrus fruit extracts	~1527	Spherical		[19]
Selenium source: Na_2SeO_3<br>Reducing/Stabilizing agent: *Coccinia grandis* fruit extract.	~27	Spherical		[20]
Selenium source: Na_2SeO_3<br>Reducing agent: Vc<br>Stabilizing agent: *Astragalus* polysaccharide.	~49	Spherical	-37	[21]
Reducing Agent: Vc<br>Selenium source: Na_2SeO_3<br>Stabilizing agent: *Sargassum fusiforme* polysaccharide, tween-80	~44	Spherical	/	[22]
Selenium source: Na_2SeO_3<br>Reducing agent: Vc<br>Stabilizing agent: Dandelion polysaccharide. Tween-80	~50	Spherical	-13.2	[23]
Selenium source: Na_2SeO_3<br>Reducing agent: Vc<br>Stabilizing agent: Hawthorn polysaccharide	138	Spherical	-18.3	[24]
Selenium source: Na_2SeO_3<br>Reducing agent: Vc<br>Stabilizing agent: Green tea nano-aggregates.	75	Spherical	-41.5	[25]

Table S4. The synthesis conditions and characteristics of SeNPs by other method.

Synthesis conditions	Size (nm)	Shape	Zeta potential (mV)	References
Selenium source：SeO_2<br>Reducing agent: Gamma irradiation<br>Stabilizer (chemical): Chitosan, sodium alginate, citrus pectin<br>Stabilizer (biological): Aqueous extract of fermented fenugreek powder by *Pleurotus ostreatus*<br>Optimal gamma dose：60 kGy, 15 kGy	~27<br>~7	Spherical	/	[26]
Selenium source: Na_2SeO_3<br>Reducing/ Stabilizing agent：Ethanol extract of fermented sugar-cane bagasse	~46	Spherical	-24	[27]

References

1. Haro-Poniatowski, E.; Escobar-Alarcón, L.; Hernández-Pozos, J.L.; Mendoza-Luna, L.G.; Guarin, C.A. Synthesis and characterization of selenium nanoparticles obtained by femtosecnd pulsedo laser ablation in liquid media. *Appl. Phys. A:Mater. Sci. Process.* **2022**, *128*. https://doi.org/10.1007/s00339-022-05956-5.
2. Geoffrion, L.D.; Hesabizadeh, T.; Medina-Cruz, D.; Kusper, M.; Taylor, P.; Vernet-Crua, A.; Chen, J.; Ajo, A.; Webster, T.J.; Guisbiers, G. Naked selenium nanoparticles for antibacterial and anticancer treatments. *ACS Omega* **2020**, *5*, 2660-2669. https://doi.org/10.1021/acsomega.9b03172.
3. Yang, Z.; Zuo, Y.; Dai, L.; Zhang, L.; Yu, Y.; Zhou, L. Effect of ultrasonic-induced selenium crystallization behavior during selenium reduction. *Ultrason. Sonochem.* **2023**, *95*, 106392. https://doi.org/10.1016/j.ultsonch.2023.106392.
4. Mellinas, C.; Jimenez, A.; Garrigos, M.D.C. Microwave-Assisted greensynthesis and antioxidant activity of selenium nanoparticles using theobroma cacao L. bean shell extract. *Molecules* **2019**, *24*, 4048 https://doi.org/10.3390/molecules24224048.
5. Yu, B.; You, P.; Song, M.; Zhou, Y.; Yu, F.; Zheng, W. A facile and fast synthetic approach to create selenium nanoparticles with diverse shapes and their antioxidation ability. *New J. Chem.* **2016**, *40*, 1118-1123. https://doi.org/10.1039/c5nj02519b.
6. Boroumand, S.; Safari, M.; Shaabani, E.; Shirza, M.; Faridi-Majidi, R. Selenium nanoparticles: synthesis, characterization and study of their cytotoxicity, antioxidant and antibacterial activity. *Mater. Res. Express* **2019**, *6*. https://doi.org/10.1088/2053-1591/ab2558.
7. Jiang, F.; Cai, W.; Tan, G. Facile synthesis and optical properties of small selenium nanocrystals and nanorods. *Nanoscale Res. Lett.* **2017**, *12*, 401. https://doi.org/10.1186/s11671-017-2165-y.
8. El-Megharbel, S.M.; Al-Salmi, F.A.; Al-Harthi, S.; Alsolami, K.; Hamza, R.Z. Chitosan/Selenium nanoparticles attenuate diclofenac sodium-induced testicular toxicity in male rats. *Crystals* **2021**, *11*, 1477. https://doi.org/10.3390/cryst11121477.
9. Chen, Y.Y.; Stoll, S.; Sun, H.B.; Liu, X.N.; Liu, W.; Leng, X.J. Stability and surface properties of selenium nanoparticles coated with chitosan and sodium carboxymethyl cellulose. *Carbohydr. Polym.* **2022**, *278*, 118859. https://doi.org/10.1016/j.carbpol.2021.118859.
10. Song, X.X.; Chen, Y.Y.; Sun, H.B.; Liu, X.N.; Leng, X.J. Physicochemical stability and functional properties of selenium nanoparticles stabilized by chitosan, carrageenan, and gum Arabic. *Carbohydr. Polym.* **2021**, *255*, 117379. https://doi.org/10.1016/j.carbpol.2020.117379.
11. Ge, M.; Zhou, S.; Li, D.; Song, D.; Yang, S.; Xu, M. Reduction of selenite to selenium nanoparticles by highly selenite-tolerant bacteria isolated from seleniferous soil. *J. Hazard. Mater.* **2024**, *472*, 134491. https://doi.org/10.1016/j.jhazmat.2024.134491.
12. Faramarzi, S.; Anzabi, Y.; Jafarizadeh-Malmiri, H. Nanobiotechnology approach in intracellular selenium nanoparticle synthesis using Saccharomyces cerevisiae-fabrication and characterization. *Arch. Microbiol.* **2020**, *202*, 1203-1209. https://doi.org/10.1007/s00203-020-01831-0.
13. Shi, M.H.; Deng, J.; Min, J.Y.; Zheng, H.Y.; Guo, M.P.; Fan, X.L.; Cheng, S.Y.; Zhang, S.P.; Ma, X.L. Synthesis, characterization, and cytotoxicity analysis of selenium nanoparticles stabilized by Morchella sextelata polysaccharide. *Int. J. Biol. Macromol.* **2023**, *242*, 125143. https://doi.org/10.1016/j.ijbiomac.2023.125143.
14. Duan, Y.H.; Li, M.J.; Zhang, S.S.; Wang, Y.D.; Deng, J.Y.; Wang, Q.; Yi, T.; Dong, X.X.; Cheng, S.Y.; He, Y.; et al. Highly efficient biotransformation and production of selenium nanoparticles and polysaccharides using potential probiotic bacillus subtilis T5. *Metabolites* **2022**, *12*, 1204-1204 https://doi.org/10.3390/metabo12121204.
15. Li, Z.J.; Wang, Q.Q.; Dai, F.J.; Li, H.F. Reduction of selenite to selenium nanospheres by Se(IV)-resistant lactobacillus paralimentarius JZ07. *Food Chem.* **2022**, *393*, 133385. https://doi.org/10.1016/j.foodchem.2022.133385.
16. Mohammed, E.J.; Abdelaziz, A.E.M.; Mekky, A.E.; Mahmoud, N.N.; Sharaf, M.; Al-Habibi, M.M.; Khairy, N.M.; Al-Askar, A.A.; Youssef, F.S.; Gaber, M.A.; et al. Biomedical promise of aspergillus flavus-biosynthesized selenium nanoparticles: A green

synthesis approach to antiviral, anticancer, anti-Biofilm, and antibacterial applications. *Pharmaceuticals* **2024**, *17*. https://doi.org/10.3390/ph17070915.

17. Cui, Y.H.; Li, L.L.; Zhou, N.Q.; Liu, J.H.; Huang, Q.; Wang, H.J.; Tian, J.; Yu, H.Q. *In vivo* synthesis of nano-selenium by Tetrahymena thermophila SB210. *Enzyme Microb. Technol.* **2016**, *95*, 185-191. https://doi.org/10.1016/j.enzmictec.2016.08.017.
18. Alvi, G.B.; Iqbal, M.S.; Ghaith, M.M.S.; Haseeb, A.; Ahmed, B.; Qadir, M.I. Biogenic selenium nanoparticles (SeNPs) from citrus fruit have anti-bacterial activities. *Sci. Rep.* **2021**, *11*, 4811-4811 https://doi.org/10.1038/s41598-021-84099-8.
19. Salem, M.F.; Abd-Elraoof, W.A.; Tayel, A.A.; Alzuaibr, F.M.; Abonama, O.M. Antifungal application of biosynthesized selenium nanoparticles with pomegranate peels and nanochitosan as edible coatings for citrus green mold protection. *J. Nanobiotechnol.* **2022**, *20*, 182. https://doi.org/10.1186/s12951-022-01393-x.
20. Jeevanantham, V.; Tamilselvi, D.; Rathidevi, K.; Bavaji, S.R. Greener microwave synthesized Se nanospheres for antioxidant, cell viability, and antibacterial effect. *J. Mater. Res.* **2023**, *38*, 1909-1918. https://doi.org/10.1557/s43578-023-00965-3.
21. Ji, H.Y.; Lou, X.W.; Jiao, J.S.; Li, Y.; Dai, K.Y.; Jia, X.Y. Preliminary structural characterization of selenium nanoparticle composites modified by astragalus polysaccharide and the cytotoxicity mechanism on liver cancer cells. *Molecules* **2023**, *28*, 1561-1561. https://doi.org/10.3390/molecules28041561.
22. Chen, Y.; Zhu, F.; Chen, J.; Liu, X.; Li, R.; Wang, Z.; Cheong, K.-L.; Zhong, S. Selenium nanoparticles stabilized by Sargassum fusiforme polysaccharides: Synthesis, characterization and bioactivity. *Int. J. Biol. Macromol.* **2024**, *269*, 132073. https://doi.org/10.1016/j.ijbiomac.2024.132073.
23. Zhang, S.J.; Song, Z.T.; Shi, L.J.; Zhou, L.A.; Zhang, J.; Cui, J.L.; Li, Y.H.; Jin, D.Q.; Ohizumi, Y.; Xu, J.; et al. A dandelion polysaccharide and its selenium nanoparticles: Structure features and evaluation of anti-tumor activity in zebrafish models. *Carbohydr. Polym.* **2021**, *270*, 118365. https://doi.org/10.1016/j.carbpol.2021.118365.
24. Sun, J.R.; Li, J.L.; Yao, L.L.; You, F.F.; Yuan, J.F.; Wang, D.H.; Gu, S.B. Synthesis, characterization and antioxidant activity of selenium nanoparticle decorated with polysaccharide from hawthorn. *J. Food Meas. Charact.* **2023**, 6125-6134. https://doi.org/10.1007/s11694-023-02124-y.
25. Ye, X.; Chen, Z.; Zhang, Y.; Mu, J.; Chen, L.; Li, B.; Lin, X. Construction, characterization, and bioactive evaluation of nano-selenium stabilized by green tea nano-aggregates. *LWT Food Sci. Technol.* **2020**, *129*, 109475. https://doi.org/10.1016/j.lwt.2020.109475.
26. El-Batal, A.I.; Mosallam, F.M.; Ghorab, M.M.; Hanora, A.; Gobara, M.; Baraka, A.; Elsayed, M.A.; Pal, K.; Fathy, R.M.; Abd Elkodous, M.; et al. Factorial design-optimized and gamma irradiation-assisted fabrication of selenium nanoparticles by chitosan and Pleurotus ostreatus fermented fenugreek for a vigorous in vitro effect against carcinoma cells. *Int. J. Biol. Macromol.* **2020**, *156*, 1584-1599. https://doi.org/10.1016/j.ijbiomac.2019.11.210.
27. El-Sayed, E.R.; Abdelhakim, H.K.; Ahmed, A.S. Solid-state fermentation for enhanced production of selenium nanoparticles by gamma-irradiated Monascus purpureus and their biological evaluation and photocatalytic activities. *Bioprocess. Biosyst. Eng.* **2020**, *43*, 797-809. https://doi.org/10.1007/s00449-019-02275-7.
